# Evasins: Therapeutic Potential of a New Family of Chemokine-Binding Proteins from Ticks

**DOI:** 10.3389/fimmu.2016.00208

**Published:** 2016-06-07

**Authors:** Pauline Bonvin, Christine A. Power, Amanda E. I. Proudfoot

**Affiliations:** ^1^Geneva Research Centre, Merck Serono S.A., Geneva, Switzerland; ^2^Research Department, Novimmune S.A., Plan-les-Ouates, Switzerland

**Keywords:** chemokines, binding proteins, antagonists, pathogens, ticks, protein therapeutics

## Abstract

Blood-sucking parasites, such as ticks, remain attached to their hosts for relatively long periods of time in order to obtain their blood meal without eliciting an immune response. One mechanism used to avoid rejection is the inhibition of the recruitment of immune cells, which can be achieved by a class of chemokine-binding proteins (CKBPs) known as Evasins. We have identified three distinct Evasins produced by the salivary glands of the common brown dog tick, *Rhipicephalus sanguineus*. They display different selectivities for chemokines, the first two identified show a narrow selectivity profile, while the third has a broader binding spectrum. The Evasins showed efficacy in animal models of inflammatory disease. Here, we will discuss the potential of their development for therapeutic use, addressing both the advantages and disadvantages that this entails.

## Introduction

The recruitment of immune cells is essential for the establishment of an immune response that, if uncontrolled, can lead to an unwanted inflammatory situation. The pharmaceutical industry has, for several decades, sought to inhibit excessive leukocyte recruitment by interference with the chemokine system, unfortunately with only limited success to date. Therefore, we investigated the manner in which pathogens and parasites avoid rejection by an immune response. It has been known for some time that pathogens, such as viruses, have pirated many molecules of the mammalian immune system, including molecules that either mimic or inhibit chemokines and their receptors to subvert the immune system. Among these molecules, there are a number of chemokine-binding proteins (CKBPs) that directly interact with chemokines to inhibit their activity (1). The vast majority of CKBPs have been identified in viruses, and these proteins are often able to recognize a large number of chemokines. As an example, M3, a protein encoded by the murine gammaherpesvirus-68, binds to chemokines from the four different subfamilies (CC, CXC, C, and CX_3_C) (2), whereas gG (from herpes simplex virus) and Crm (encoded by smallpox virus) interact with chemokines from at least three subfamilies (3, 4). In 2005, the first eukaryotic CKBP was identified in the worm *Schistosoma mansoni* and was shown to bind promiscuously to some CC chemokines, notably CCL3 and CCL5, CXCL8, and CX3CL1 *in vitro* (5).

Following a report describing anti-CXCL8 activity in the salivary glands of several Ixodid tick (or hard tick) species (6), we extended this observation by identifying the molecule responsible for this activity. To do this, we first tested the ability of the saliva from the hard tick *Rhipicephalus sanguineus* to inhibit the binding of three chemokines, CCL3, CCL5, and CXCL8 to their receptors, and were also able to identify the presence of these molecules in the saliva by surface plasmon resonance (SPR) and mass spectrometry (7). Using cDNA expression libraries constructed from the tick salivary glands, we used a cross-linking approach to analyze the proteins secreted into culture supernatants after transient expression of pools of cDNAs in HEK293 cells. We successfully identified three CKBPs that we named the Evasins (7, 8).

## Characteristics

Evasins have been quite extensively characterized in terms of their chemokine-binding profile and inhibitory potency *in vitro* and *in vivo* activity. On the one hand, Evasin-1 demonstrated the highest specificity as it binds only to three closely related chemokines, CCL3, CCL4, and CCL18. On the other hand, Evasin-3 recognizes a subset of CXC chemokines, the family of the so-called ELR^+^ chemokines, i.e., CXCL1, -2, -3, -5, -6, and -8. Both of these CKBPs efficiently inhibit the activity of their ligands, preventing cell migration *in vitro*. Evasin-4, which was initially identified using I^125^-labeled CCL5 and CCL11 as bait, displayed a broader selectivity profile than the other two Evasins, being able to interact with at least 18 chemokines, yet it is still highly specific as it recognizes only members of the CC subfamily (9). Although minor discrepancies were observed between the binding and the inhibitory profile of Evasin-4, it blocks the activity of the majority of its ligands, including the proinflammatory chemokines CCL3, 5, 8, and 11.

Based on their binding profile, Evasin-1 and -3 would be expected to inhibit the migration of neutrophils in mice, which is crucial in the first steps of the immune response. Here, it should be noted that one of the major differences in the leukocyte recruitment profiles in the chemokine system is that of the neutrophil. In mice, neutrophils express both CCR1 as well as CXCR2, which results in the recruitment of this leukocyte by both the CCL ligands and the CXCL ligands activating these receptors, respectively. Regarding Evasin-4, its ability to prevent the interaction of CC chemokines with their receptors may also lead to the inhibition of eosinophil recruitment, an essential cellular component of the response against parasites.

Vancova et al. have reported the presence of anti-CXCL8 activity and anti-CXCL1 activity in salivary gland extracts from males and females of several other species of ticks: *Amblyomma variegatum*, *Rhipicephalus appendiculatus*, and *Dermacentor reticulatus*, suggesting that Evasin-3-like activity is common among metastriate ixodid tick species (10). In a separate study, the same group also reported activity against human CXCL8, CCL2, CCL3, CCL5, and CCL11 in adult *R. appendiculatus* ticks (11), suggesting that Evasin-1 and Evasin-4 orthologs probably exist in this species too. Also in this study, the authors showed that anti-chemokine activity differed significantly at different times during feeding and also differed between males and females supporting the concept of “mate guarding,” in which males help their mates to engorge by controlling their host’s immune response, and the possibility that ticks benefit from feeding together in close proximity by exploiting molecular individuality. Interestingly, in this species, anti-CCL11 activity was high in unfed ticks, initially declined, and then increased in both males and females as feeding progressed (11).

As previously mentioned, the existence of viral CKBPs was reported before the identification of Evasins. However, although they probably share similar functions *in vivo*, several differences have been highlighted between these two families of CKBP. First, as mentioned above, the large majority of viral CKBPs display broad-spectrum chemokine-binding profiles, whereas the Evasins are much more selective. It is noteworthy that the Evasin-4-binding profile closely mimics that of the viral CKBP vCCI. These two CKBPs recognize between 13 and 18 chemokines, yet are still highly selective in that they bind only to CC chemokines (9, 12). Therefore, among CKBPs, they form a unique class of chemokine binders with “semi-broad” selectivity.

Another key difference between tick and viral CKBPs is the size of these proteins. Viral CKBPs are large proteins, which might even form dimers as reported for M3 (13). On the contrary, Evasins are small proteins of around 80–100 amino acids, indicating that the two partners of the chemokine:Evasin interaction have similar sizes.

## Do Evasins Exist in Other Species?

We were intrigued to know whether these molecules formed a family of CKBPs in both ticks and in other species, particularly man. Blasting their sequences against the human genome, and all other mammalian genomes available, did not produce any significant hits.

However, at least five putative Evasin-1 and Evasin-3 homologs have been identified following in-depth sequence analysis of the *R. sanguineus* sialotranscriptome (14). Expressed sequence tags that are Evasin-3-like have been identified in *Ixodes scapularis*, *Ixodes ricinus*, and *Dermacentor andersoni* (10), and the sequences of potential Evasin homologs have also been identified in Genbank for *Boophilus microplus* and *I. scapularis* (Power, unpublished analysis). At least 18 Evasin homologs have been identified for *Amblyomma maculatum* (15), and Radulovic et al. recently reported a sequence homologous to Evasin-1 in *Amblyomma americanum* (16). Considering there are over 700 Ixodidae species, and about 200 soft tick or Argasidae species, with recent developments in next generation sequencing and proteomics, it is likely that many more homologous sequences will be identified in the coming years. Yet whether any of the homologs described above encode a CKBP has not yet been confirmed by functional analysis.

**Figure 1:**
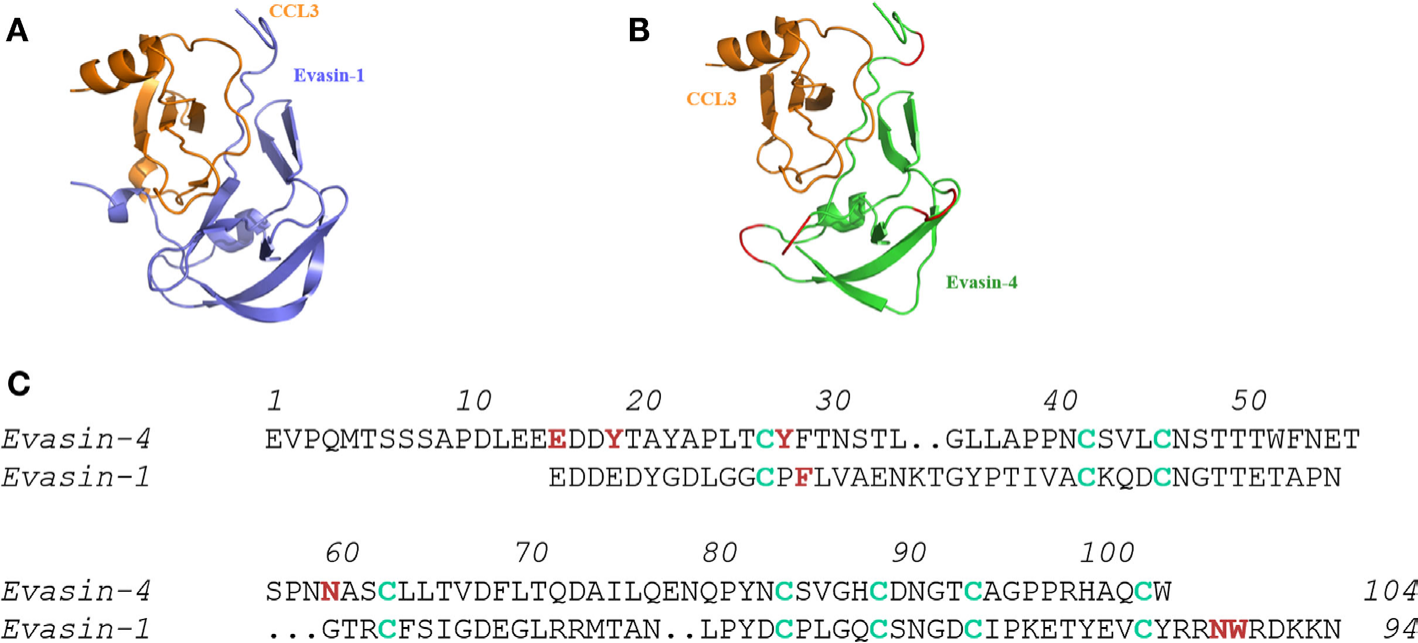
**Molecular interactions of Evasin-1 and -4 with CCL3**. **(A)** Structure of the complex of Evasin-1 and CCL3 determined by X-ray crystallography. **(B)** Evasin-4 in complex with CCL3 by *in silico* modeling using **(A)** (17). **(C)** Alignment of the primary amino acid sequences of Evasin-1 and -4. Cys residues are shown in green and amino acids identified to play a role in chemokine binding are shown in red (17), demonstrating that the selective CHBP, Evasin-1 predominantly uses the carboxy terminal region, whereas Evasin-4 that binds many CC chemokines predominantly uses the amino terminal region.

Even though blasting the primary sequences of the Evasins did not reveal homologs in mammals, we hypothesized that proteins with similar folds might exist in eukaryotics. While the structures of Evasin-1 and Evasin-3 were found to be totally different from each other, blasting the PDB database of three-dimensional protein structures, once again did not produce any hits. The third CKBP, Evasin-4, has a disulfide bridge pattern that aligned with that of Evasin-1, indicating that it would probably have the same three-dimensional fold (Figure 1) (17), so it was unlikely to possess a different structural motif. Thus, it appears that while this tick species has unique CKBPs, one cannot rule out that other ticks, particularly hard ticks that feed for extended periods, will have their own unique CKBP(s).

## Activity *In Vivo*

The Evasins have all shown efficacy *in vivo* in several disease models. As predicted from its binding profile, Evasin-1 reduced neutrophil recruitment induced by CCL3 in a peritoneal cell recruitment assay in a dose-dependent manner (8). This highlights one of the anomalies of translating *in vivo* data from mice to man as described above. In humans, CCL3 is not a principal mediator of neutrophil recruitment since its receptors CCR1 and CCR5 are primarily expressed on monocytes/macrophages, although they can be induced, for example, by IFNγ (18). However, in mice, CCR1 is highly expressed on neutrophils, resulting in strong recruitment in response to CCL3. On the other hand, neutrophil recruitment in mice is also mediated by CXCR2 ligands, as it is in the human system. Thus, in accordance with its ability to inhibit neutrophil infiltration, Evasin-1 showed good efficacy in reducing the fibrosis, which follows neutrophil infiltration into the lung after bleomycin administration, and also reduced the mortality observed in this model (19).

Evasin-3 was effective in several neutrophil-dependent disease models as expected from its *in vitro* selectivity profile, showing that it binds and inhibits ELR^+^ chemokines that bind to CXCR2. Again, dose-dependent inhibition of leukocyte infiltration into the peritoneal cavity, this time in response to CXCL8, was inhibited by Evasin-3. Antigen-induced arthritis (AIA), induced by intradermal administration of mouse BSA, is highly neutrophil dependent. In AIA, disease symptoms were significantly decreased by the administration of Evasin-3. In another neutrophil-mediated scenario, ischemic reperfusion injury, both Evasin-1 and Evasin-3 were effective, but Evasin-3 was shown to be more efficacious, indicating that the CXCR2 ligands play a predominant role in this model. On the contrary, only Evasin-1 and not Evasin-3 was effective in inhibiting the first wave of dendritic cell recruitment to the site of infection with *Leishmania major*, since it is mediated by neutrophil-secreted CCL3 (20). Intriguingly, despite the fact that Evasin-1 has only been shown to bind to three chemokines *in vitro*, it was able to reduce the skin inflammation observed in the D6^−/−^ mice in response to 12-*O*-tetradecanoylphorbol-13-acetate (TPA) (8), a model which had previously been shown to depend on several inflammatory ligands (21).

Studies with Evasin-4 produced some puzzling observations. In line with its broad selectivity profile and inhibitory activity against several CC chemokines known to have proinflammatory activity, it was shown to be effective in reducing post-infarction myocardial injury and remodeling (22) and DSS-induced colitis (23), yet Evasin-3 that only binds ELR^+^ CXC chemokines *in vitro* was also effective in the myocardial injury model. This highlights the problem in the translation of agents inhibiting neutrophil-mediated inflammation in mice to the human setting.

**Figure 2:**
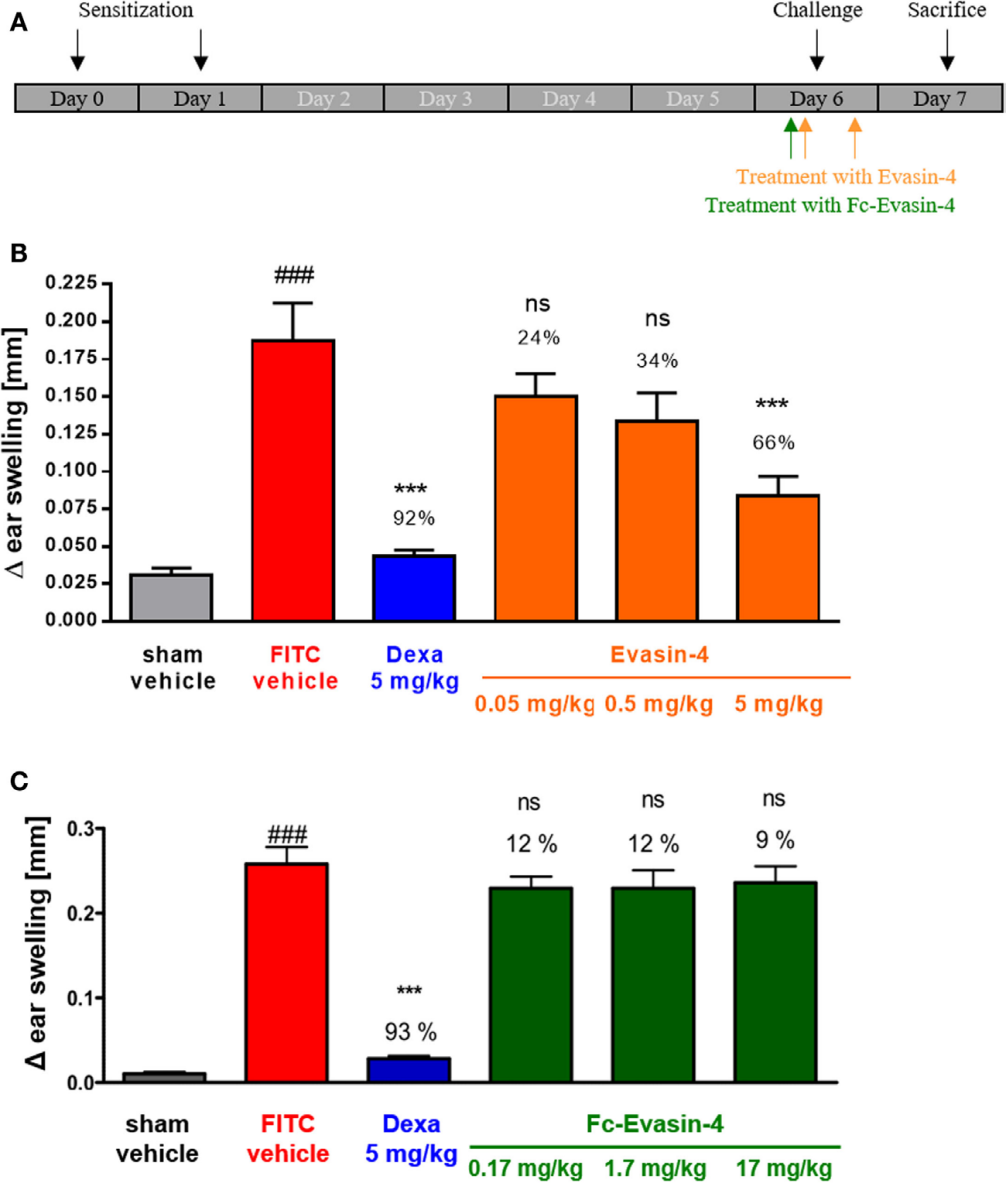
**FITC-induced contact hypersensitivity**. **(A)** Schematic design of the experiment. **(B)** Treatment with Evasin-4 reduces ear swelling (*n* = 5–9 mice per group). **(C)** Treatment with Fc-Evasin-4 does not prevent ear inflammation (*n* = 8 mice per group). Data are presented as mean ± SEM and were analyzed with one-way ANOVA and Dunnett’s post-test. The percentage of ear swelling inhibition is reported for each treatment. ^###^*p* < 0.001 compared with sham, **p < 0.01 or ***p < 0.001 compared with vehicle-treated FITC group, ns not significant.

Because of its broad CC chemokine-binding spectrum, Evasin-4 was considered the most suitable Evasin for development as a possible therapeutic candidate. However, it is well known that small proteins have a very short half-life *in vivo* and are not orally available, which means that for chronic indications, they would have to be injected with a frequency that is not convenient for patients. In order to prolong the serum half-life of therapeutic proteins, the strategy of making Fc fusions is often employed. Therefore, we created fusions of Evasin-4 with the Fc portion of human IgG1, making both N- and C-terminal versions (9). Having characterized the Fc fusion proteins *in vitro* and selected the format that had activity closest to wild-type Evasin-4, we compared their activity in a simple disease model, fluorescein isothiocyanate (FITC)-induced contact hypersensitivity. While Evasin-4 had dose-dependent efficacy in reducing the disease symptoms, molar equivalents of the fusion protein were totally ineffective (Figure 2).

The negative result obtained with Evasin 4 fused to the C-terminal of Fc (Fc-Evasin-4) was unexpected as we predicted that the Fc domain would increase the therapeutic potential of Evasin-4 and not abrogate its anti-inflammatory activity. In the hypersensitivity model, the treatment schedule was based on the half-life of the protein, and as Fc-Evasin-4 exhibited a much longer half-life than Evasin-4, Evasin-4 was injected twice, whereas Fc-Evasin-4 was injected only once, before the challenge with FITC. These different treatment schedules may explain the lack of activity of Fc-Evasin-4 in comparison to Evasin-4. Our current hypothesis is based on the fact that during inflammation, a large amount of chemokines is produced and that the administration of one single dose of the Fc fusion protein might not have been sufficient to saturate the system.

This was confirmed by the measurement of chemokine levels in serum samples from contact hypersensitivity models, which demonstrated a significant accumulation of MCP-1 (the murine equivalent to CCL2), MIP-1α (equivalent to CCL3), and RANTES (equivalent to CCL5) following treatment with Fc-Evasin-4, as has been reported for administration of an anti-CCL2 antibody (24). Therefore, although the extended half-life of Fc-Evasin-4 allows it to remain in the circulation for more than 1 week, the drug is probably rapidly saturated and no unbound Fc-Evasin-4 molecules are available to inhibit newly produced chemokines and to reduce inflammation. In the case of Evasin-4, CKBP:chemokine complexes are probably more rapidly degraded, preventing their accumulation in circulation. Furthermore, the second bolus of drug provides an additional amount of free Evasin-4 molecules available to inhibit chemokine activity. Therefore, although theoretically equimolar, the doses of Evasin-4 and Fc-Evasin-4 used are probably not equivalent *in vivo*. Thus, the Fc fusion would only be useful if the chemokine is released and degraded in the endosome allowing the fusion protein to recycle back into the circulation to pick up more chemokines, necessitating engineering the Evasin to bind chemokine at pH 7.2 and release it at pH 6.0, as has been reported for mAbs (25, 26).

## Therapeutic Potential

A major consideration for the development of protein therapeutics is immunogenicity – where the body elicits an immune response (production of antibodies) against the therapeutic entity. Antibodies against a therapeutic protein may elicit a wide range of consequences, from no detectable change to life-threatening conditions. One of the main concerns is altered drug safety or compromised efficacy. Antibody formation may attenuate the efficacy even to the extent that higher doses cannot overcome the clinical resistance induced by the antibody response. Deleterious effects can occur when antibodies against a therapeutic agent cross-react with endogenous proteins. Neutralizing the endogenous protein can be particularly dangerous, especially if it is a unique protein with non-redundant function. Such was the case for erythropoietin a few years ago. An immune response that neutralized the activity of both the administered recombinant protein Eprex^®^ and that of the endogenous protein in patients had dramatic consequences, resulting in an acute anemia called pure red-cell aplasia (PRCA) that was fatal in a few patients [reviewed in Ref. (27)]. In the case of non-human proteins, especially those with no homology to any known human protein, this type of reaction would not be relevant. Nevertheless, nearly all therapeutic proteins – be they native human proteins, monoclonal antibodies, antibody drug conjugates, or fusion proteins – can elicit an immune response. Another potential danger of antibody formation against therapeutic proteins is the elicitation of immunoglobulin (Ig) E-mediated hypersensitivity reactions ranging from local skin reactions to more severe systemic reactions such as anaphylaxis. However, cases of anaphylaxis have been seen with almost every substance administered to man, ranging from peanuts to recombinant interferon-β, but fortunately are not common.

Nevertheless, there are already examples of non-human proteins in the clinic. Hirudin, a small protein produced by leeches, is an inhibitor of thrombin and is used extensively for the retreatment of heparin-induced thrombocytopenia for patients undergoing hip replacement surgery (28). A second example is the GLP-1 receptor agonist exenatide (synthetic exendin-4), a 39 amino acid peptide, marketed as Byetta^®^, which was originally identified in the salivary secretions of a poisonous lizard known as the Gila monster (*Heloderma suspectum*). Exenatide was developed as a first-in-class type 2 diabetes therapy (29). In a recent report, it was shown that low-titer anti-exenatide antibodies were common with exenatide treatment but had no apparent effect on efficacy. Higher titer antibodies were less common, and increasing antibody titer was associated with reduced average efficacy, but other than injection-site reactions there were no safety issues (30).

A number of factors are now known to influence the immunogenicity of therapeutic proteins, but in general, the less “human” a protein is, the more likely it is to elicit an immune response, particularly following repeated administrations. However, predicting immunogenicity remains a subject of much debate. We used both proprietary (Antipred) and publicly available software (TEPITOPE) for *in silico* prediction of potential CD4^+^ T-cell epitopes in the Evasins. Interestingly, interferon-β, one of the most widely used treatments for multiple sclerosis, was predicted to contain more antigenic sites than the Evasins (unpublished data).

It should also be noted that the Evasins produced by the tick are highly glycosylated proteins. Their apparent molecular weights as estimated by SDS-PAGE analysis during their expression cloning was about five times their actual protein mass. This could be hypothesized to render them less susceptible to a host immune response. The counter argument to their status of immune-silent is that they are injected into the host in saliva containing a plethora of other proteins, which could equally play a role in preventing an immune reaction. This is obviously not the case for a therapeutic protein, where in fact the route of administration and relatively large amounts that would be administered systemically (compared to the miniscule amounts injected locally in tick saliva) would be influential on the ensuing immunogenicity.

In view of the potential of the Evasins as therapeutic modalities, we produced Evasin-3 and -4 as Fc fusion proteins. This was to counteract their rapid elimination as is the case for all small proteins. Both fusion proteins retained neutralizing activity *in vitro* comparable to the wild-type proteins. However, surprisingly, Evasin-4 lost its neutralizing activity when administered as an Fc fusion. The WT protein showed dose-related activity in inhibiting the clinical symptoms in a contact hypersensitivity model, but administration of equivalent molar amounts of the Fc fusion had no effect whatsoever. The reason for this remains unexplained, but these results prompt us to wonder whether simultaneous inhibition of chemokines with a multispecific chemokine-binding protein may be an efficient strategy to clinically improve chemokine-driven diseases. As pan-specific chemokine inhibitors bind to multiple targets, and as the amount of chemokines present in the body may be very large, due to immobilization on cell surfaces, as well as to rapid turnover and production rates, multispecific inhibitors might be saturated *in vivo*, and very high doses may be required to observe an anti-inflammatory activity. This hypothesis may also explain the lack of long-term efficacy reported with the fusion protein vCCI-Fc (31). As discussed above, a solution could be the engineering of the CKBPs to render them pH dependent. Alternatively, if simultaneous inhibition of several chemokines is required, a more successful strategy may be the broad inhibition of chemokine-induced cell migration without direct interaction with chemokines or their receptors. This strategy is exemplified by the broad-spectrum chemokine inhibitors known as somatotaxins, such as NR58-3.14.3, which is effective in a range of inflammatory disease models, including atherosclerosis and graft-versus-host disease (32–34). Another example of broad-spectrum chemokine inhibition would be by interfering with chemokine signaling as demonstrated by PI3K inhibitors. These results suggest that indirect interference with cell migration may be a promising strategy to prevent excessive recruitment to inflamed sites.

**Figure 3:**
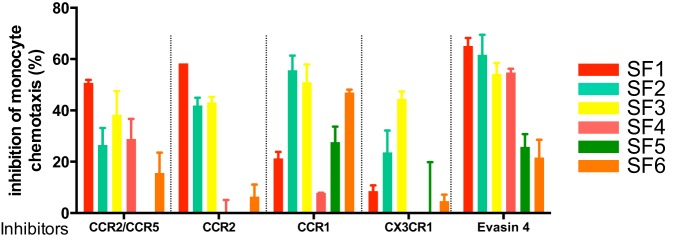
**Inhibition of synovial fluid-induced monocyte recruitment by selective chemokine receptor inhibitors and Evasin-4**.

It is clear from the above examples that certain pathologies may be driven by the action of several chemokines acting on distinct receptors, thus arguing that the use of broad-spectrum chemokine antagonists or at least multispecific antagonists would be beneficial. However, a recent report demonstrating biased agonism (35) has provided rationale for targeting individual ligands with biologicals, such as mAbs, to avoid off-target effects. A good example is the receptor CXCR3, whose ligands CXL10 and CXCL11 play opposing roles – the former having a proinflammatory activity while the latter is anti-inflammatory (36). To date, most of the therapeutic approaches taken by the pharmaceutical industry have been to inhibit individual chemokine receptors. Nevertheless, there are still gaps in our understanding of their precise roles. An interesting example is CCR2 that binds several CC chemokines: CCL7, CCL8, and CCL14, which also bind to other receptors, but the CCL2/CCR2 interaction is non-redundant. CCR2 has been targeted in three separate clinical trials without much success. Is this due to a problem with the target or the drug? The importance of the CCL2/CCR2 interaction in monocyte recruitment is compelling, yet animal models suggest that CCR1 may also play role (37). How does this relate to monocyte recruitment in human disease? In a recent *in vitro* study, we looked at the ability of specific chemokine receptor antagonists and Evasins to block monocyte chemotaxis in response to synovial fluid harvested from six rheumatoid arthritis patients (Proudfoot et al., unpublished data).^1^ We observed that only Evasin-4 could inhibit monocyte migration in all samples (Figure 3). In this system, Evasin-4 acts as a soluble chemokine receptor with specificity for multiple monocyte-directed ligands, providing a much more simple approach to chemokine antagonism than targeting one, two, or even multiple chemokine receptors with small-molecule antagonists or antibodies.

Thus, the Evasins present therapeutic potential in pathologies where several chemokines are involved. However, there are certain aspects that must be addressed for future development of these molecules. In view of the observed lack of activity with the Evasin-Fc fusion, treatment of acute indications, where short half-life is not a problem, would be first choice. Moreover, the administration of such proteins for an acute regimen would circumvent the potential issues of immunogenicity. The development of the Evasins for more chronic diseases would require optimization of the potential biologic modality, for example, half-life extension. Production of a pH-dependent chemokine-binding molecule could also solve the problem of the large amount of target protein(s) to be neutralized. With their small size and unique structure, Evasins are also very attractive targets for protein engineering to introduce exquisite specificity, as more and more information becomes available on the role of specific chemokines in human disease. However, we believe that future directions in the search for novel innovative approaches to the treatment of inflammatory diseases should include the study of how nature deals with the immune system – there is still a lot to be learnt from pathogens and parasites that have evolved elegant mechanisms to avoid rejection by their hosts.

## Author Contributions

AP initiated and directed the project and wrote the manuscript; CP supervised the cloning and characterization of Evasins and wrote the manuscript; and PB performed experiments and wrote the manuscript.

## Conflict of Interest Statement

AP, PB, and CP are former employees of Merck Serono S.A. AP and PB are former employees of Novimmune S.A.

## Footnotes

^1^A. Proudfoot, C. Gabay, A. Garin. 2016, unpublished work
